# Monitoring air pollutants in urbanized hydrothermal areas: challenges and benefits of traditional measurement strategies

**DOI:** 10.1007/s10653-025-02422-y

**Published:** 2025-03-16

**Authors:** Leonardo Fantini, Stefania Venturi, Francesco Capecchiacci, Tullio Ricci, Rebecca Biagi, Franco Tassi

**Affiliations:** 1 Department of Earth Sciences, University of Florence, Via G. La Pira 4, 50121 Florence, Italy; 2 Institute of Geosciences and Earth Resources (IGG), National Research Council of Italy (CNR), Via G. La Pira 4, 50121 Florence, Italy; 3 Istituto Nazionale di Geofisica e Vulcanologia, Sezione di Palermo, Via Ugo La Malfa 153, 90146 Palermo, Italy; 4 Istituto Nazionale di Geofisica e Vulcanologia, Sezione di Napoli, Osservatorio Vesuviano, Via Diocleziano 328, 80122 Naples, Italy; 5 Istituto Nazionale di Geofisica e Vulcanologia, Sezione di Roma1, Via di Vigna Murata 605, 00143 Rome, Italy

**Keywords:** Air quality, Greenhouse gases, Hydrothermal fluid, Air monitoring, Anthropogenic impact

## Abstract

Carbon- and sulfur-bearing gases are emitted at relevant amounts from hydrothermal manifestations at Tivoli Terme (Latium, central Italy), where different potential anthropogenic contaminant sources also occur. This study presents a geochemical dataset including CO_2_, CH_4_, SO_2,_ and H_2_S concentrations and δ^13^C-CO_2_ and δ^13^C-CH_4_ values measured in air at the center of Tivoli town. The main aim was to evaluate the impact on air quality of hydrothermal manifestations and anthropogenic activities employing a traditional stationary monitoring strategy. The analytical results reveal significant air contamination from both natural and anthropogenic emissions, although gas levels were below outdoor air quality thresholds. Carbon dioxide and CH_4_ were primarily linked to anthropogenic sources, while hydrothermal emissions played a secondary role. However, H_2_S concentrations up to 282 ppb highlighted a notable impact from hydrothermal emissions surrounding the measurement station, where SO_4_-rich pools are located. Nevertheless, the geochemical data did not provide a reliable estimate of the specific contributions from each source. The study identifies key limitations in relying on a single fixed monitoring station, as weather conditions highly influence it and cannot reliably capture the relative impacts of various sources across a broad area. Additionally, compositional and isotopic geochemical parameters often produce ambiguous results, complicating the differentiation of pollution sources. An integrated approach is recommended, combining mobile stations for periodic pollutant mapping with low-cost instruments deployed at strategic locations near potential sources and progressively farther away. This strategy could better track the spatial and temporal evolution of contaminant concentrations, addressing the shortcomings of current monitoring systems and enhancing mitigation efforts.

**Supplementary Information:**

The online version contains supplementary material available at 10.1007/s10653-025-02422-y.

## Introduction

Hydrothermal systems in Italy, which are mostly associated with current or past volcanic activity (e.g. Campi Flegrei, Tuscan Roman Degassing Structure, Campanian Degassing Structure), are situated in urbanized areas inhabited by thousands up to millions of people (e.g. Chiodini et al., 2001; Cardellini et al., 2017). In these areas, pollutant gases emitted into the atmosphere from natural sources add to anthropogenic air contaminants typically characterizing the urbanized environments. On a global scale, air pollution from hydrothermal systems worldwide cannot be compared to the amount originating from anthropogenic sources, but such a natural contaminant source remains substantial and potentially hazardous for human health at a local scale. Specifically, 83 × 10^12^ g/yr of CO_2_ and 23 × 10^12^ g/yr of SO_2_ are estimated to be globally released by volcanoes through passive degassing (Carn et al., 2016; Werner et al., 2019). Carbon dioxide, typically found at anomalous concentrations in urban environments being produced by different anthropogenic activities, is one of the major greenhouse gases (Bikis, 2023; Hoornweg et al., 2011). On the other hand, SO_2_ and H_2_S react with hydroxyl radicals (OH) and water vapor in the air to form H_2_SO_4_, which creates an aerosol capable of reflecting solar radiation, leading to widespread cooling of the troposphere and warming of the stratosphere (McCormick et al., 1995; Rampino & Self, 1982; Self et al., 1993). Sulfur volatiles can also contribute to the degradation of the ozone layer and the production of acid rain (Andres & Kasgnoc, 1998; McGee et al., 1997; Robock, 2000; Textor et al., 2003; Von Glasow et al., 2009). Additionally, SO_2_ is irritant to the lungs and, even at low concentrations, could cause inflammation of the eyes and respiratory tract (Sax & Lewis, 1989).

Continuous monitoring of these gases is crucial in urban centers situated in hydrothermal areas to evaluate the impact on air quality of both (i) Natural and (ii) Anthropogenic emissions. Several studies demonstrated that multiparametric approaches that combine compositional and isotopic data and meteorological parameters are successful in identifying different types of contributions in multisource contexts (e.g., Bezyk et al., 2023; Biagi et al., 2022; Carapezza et al., 2003, 2015, 2020; Górka et al., 2014; Lopez et al., 2017; Lowry et al., 2001; Minissale et al., 1997; Tassi et al., 2012; Townsend-Small et al., 2012; Venturi et al., 2020, 2021; Widory & Javoy, 2003; Zimnoch et al., 2019). In the surroundings of the Alban Hills volcanic complex, a quiescent system situated in a densely inhabited area east of Rome (central Italy), hydrothermal emissions strongly contribute to affect air quality (Annunziatellis et al., 2003; Carapezza & Tarchini, 2007; Venturi et al., 2019). At Cava dei Selci village, for example, CO_2_ discharged to the atmosphere reaches values up to 95.7 × 10^6^ g/d, i.e. the same order of magnitude as the CO_2_ emissions estimated for active volcanoes in southern Italy such as Stromboli (Carapezza & Federico, 2000) and Vulcano (Chiodini et al., 1996). Not far from this site, at Tivoli town, hosting a population of more than 50,000 inhabitants, an intense hydrothermal activity occurs (Brunetti et al., 2013; La Vigna et al., 2015), as testified by the extensive travertine deposits currently under exploitation (e.g. De Filippis, 1998; De Filippis et al., 2011, 2013). The present study presents a geochemical dataset including CO_2_, CH_4_, SO_2_ and H_2_S concentrations and δ^13^C-CO_2_ and δ^13^C-CH_4_ values measured in air at the center of Tivoli during four campaigns carried out from August 4th to September 2nd, 2021. The main aim is to evaluate the impact on air quality of (i) Hydrothermal manifestations and, separately, (ii) Anthropogenic activities, while assessing the benefits and limitations of using a single stationary monitoring station, as traditionally employed by environmental agencies in multisource air pollution areas.

## Study area

**Fig. 1:**
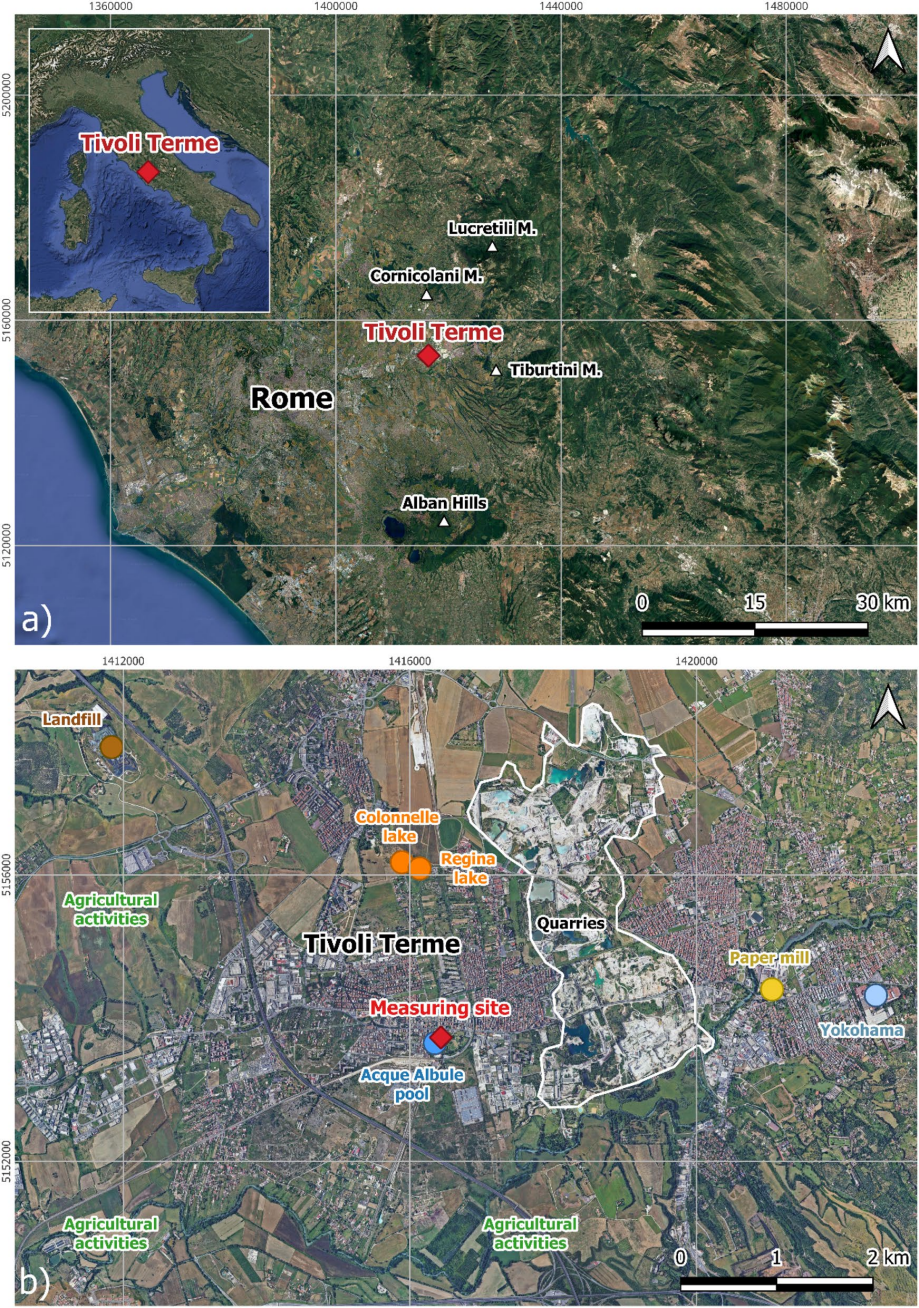
**a** Satellite image of the Latium area with location of Tivoli Terme, main mountains of the Apennine chain, and the caldera rim of the volcanic district of Alban Hills **b** Location of the measuring site and the potential sources of the analyzed gases in Tivoli Terme

Tivoli town (Latium, Italy) is located within the Acque Albule Basin (AAB), on the western margin of the Central Apennines (Argentieri et al., 2015) (Fig. 1a). The AAB, approximately 20 km east of Rome, covers an area of about 30 km^2^ and is bordered to the north and east by meso-cenozoic marine carbonate deposits of the Cornicolani, Lucretili, and Tiburtini Mountains (forming the Apennine chain) (Fig. 1a). To the south, it is bordered by the volcanic district of the Alban Hills, whose caldera rim is just 18 km away (Fig. 1a). This region is affected by significant subsidence phenomena that have led to the formation of the Regina and Colonnelle lakes that are mostly fed by thermal springs (Bianchini et al., 2024; Billi et al., 2016; Ciotoli et al., 2015; Nisio, 2008) (Fig. 1b). All the thermal springs discharging in the southern sector of the AAB have a Ca-HCO_3_(SO_4_) composition due to interaction with Mesozoic rock of the Tuscan stratigraphic series that is the main hydrothermal reservoir (Annunziatellis et al., 2003; Carapezza & Tarchini, 2007; De Filippis, 1998; La Vigna et al., 2015; Minissale, 2004). The extensive plate of thermogenic travertine that fills the Acque Albule Basin, under intensive exploitation since ancient times (Billi et al., 2016; Faccenna et al., 2008; ISPRA, 2017), has been produced by CaCO_3_ precipitation at the emergence of oversaturated thermal waters.

The measurement site for air quality monitoring of the present investigation was located on the roof of the “Acque Albule” thermal complex, in the southern part of the urban area of Tivoli Terme (Fig. 1b). In the proximity of the measurement site several potential natural sources of C- and S-bearing volatiles occur, as follows: (i) Regina and Colonnelle lakes (2 km to the N) (ii) The Acque Albule pools (few tens of meters to the S-SW), and (iii) ponds and small artificial lakes within the travertine quarries (2 km to the NE-E) (Fig. 1b). Potential air contaminant sources related to anthropogenic activities include: (i) Vehicular traffic from Tivoli Terme (to the N), (ii) Areas designated to intense agriculture and livestock (mostly located to the W and S), (iii) The Inviolata landfill, (4 km to the NW), (iv) A paper mill (3 km to the E), and (v) A tire manufacturing industry (Yokohama) (4 km to the E) (Fig. 1b).

## Materials and methods

### Measurement strategy, instrumental equipment and data acquisition and analysis

Carbon-bearing pollutants in air were measured in 2021 during four surveys lasting from (i) August 4th to 7th, (ii) August 20th to 21st, (iii) August 28th to 29th, and (iv) August 31st to September 2nd. Hydrogen sulfide and SO_2_ concentrations were measured from August 4th to 7th and from August 20th to 21st.

Carbon dioxide and CH_4_ concentrations (in ppm) and the δ^13^C–CO_2_ and δ^13^C–CH_4_ values (in ‰ vs. V-PDB) were measured by WS-CRDS (Wavelength-Scanned Cavity Ring-Down Spectroscopy) using a Picarro G2201-*i* analyzer with an operating interval ranging from 380 to 2,000 ppm for CO_2_, and from 1.8 to 15 ppm for CH_4_ (high-precision mode). The precision was within 0.22 ppm (CO_2_), 0.05 ppm (CH_4_), 0.16 ‰ (V-PDB) (δ^13^C–CO_2_) and 1.15 ‰ (V-PDB) (δ^13^C–CH_4_) (Biagi et al., 2022). Hydrogen sulfide and SO_2_ concentrations (in ppb) were measured by PF (Pulsed Fluorescence) using a Thermo® 450*i* analyzer (Thermo Fisher Scientific) with a detection limit of 2 ppb for SO_2_ and H_2_S, and a precision of ± 1% (Venturi et al., 2016). Air samples were drawn through Teflon tubing using vacuum pumps with sampling rates of 25 and 70 mL min^−1^ for the Picarro and the Thermo, respectively. Both instruments provided one measurement per minute. Meteorological parameters were provided by "Meteo Lazio" (www.meteoregionelazio.it), which is a meteorological center dedicated to the Latium region, integrated with those available online at www.wunderground.com and www.weatherspark.com.

## Results

### Meteorological parameters

The measurement surveys were characterized by sunny weather conditions except on August 20th and 28th when thunderstorms (up to 23.5 mm of rainfall) occurred. Meteorological data (air temperature, wind speed and direction) are reported in the Supplementary Material (Table S1). Air temperatures ranged from 14.8 °C to a maximum of 34.3 °C. In the first period (August 4th to 7th), prevailing winds came from SW and S with speeds ranging between 15 and 30 km/h during the daytime, whereas during the nighttime the wind direction ranged from WSW to SW and wind speed did not exceed 10 km/h. On August 20th-21st, wind mainly flew from the northern sector with a speed up to 15 km/h during the day and up to 10 km/h in the night. Winds had a similar direction during the third and the fourth surveys (August 28th to 29th and August 31st to September 2nd, respectively) with speeds up to 20 km/h.

### Gas concentrations and δ^13^C values

**Fig. 2:**
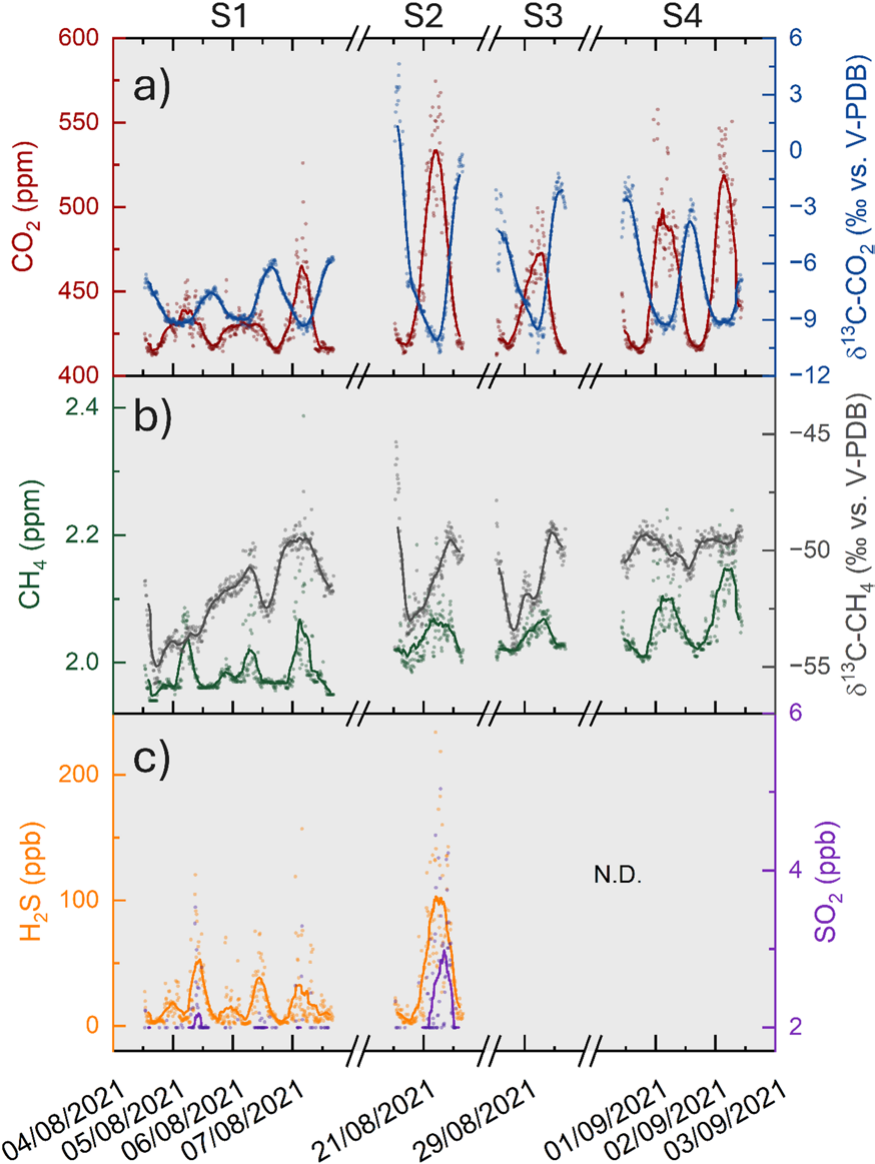
Temporal evolution of ten-minute averages of **a** CO_2_ concentrations and δ^13^C-CO_2_ values, **b** CH_4_ concentrations and δ^13^C-CH_4_ values, and **c** H_2_S and SO_2_ concentrations (points) during the four surveys (S1-4). Thirty-minute moving average values are also shown as continuous lines

Ten-minute average concentrations of CO_2_, CH_4_, SO_2_, and H_2_S and the δ^13^C-CO_2_ and δ^13^C-CH_4_ values measured during the surveys are reported in Figs. 2a, b, c, respectively. Thirty-minute moving average trends are also shown as continuous lines (Figs. 2a, b, c).

The CO_2_ concentration ranged from 412 to 575 ppm, whereas the δ^13^C-CO_2_ values ranged from -10.8‰ to + 4.6‰ vs. V-PDB. At a first approximation, these two parameters were inversely correlated, and both showed the largest variations during the second measurement period (August 20th to 21st), whereas during the first measurement period (August 4th to 7th) their range of variation was relatively low.

The CH_4_ concentrations ranged from 1.94 to 2.39 ppm, and the δ^13^C-CH_4_ values ranged from − 55.7‰ to − 45.3‰ vs. V-PDB. The temporal evolution of the two parameters did not show any significant correlation (Fig. 2b), whereas the first measurement period was characterized by relatively low values, in agreement with the CO_2_ concentrations (Fig. 2a).

The H_2_S concentrations ranged from the detection limit (2 ppb) to 234 ppb, whereas those of SO_2_ were from the detection limit (2 ppb) to 5.0 ppb. Although the concentrations of these two parameters differed by almost 2 orders of magnitude, they showed a significant correlation (Fig. 2c).

**Fig. 3:**
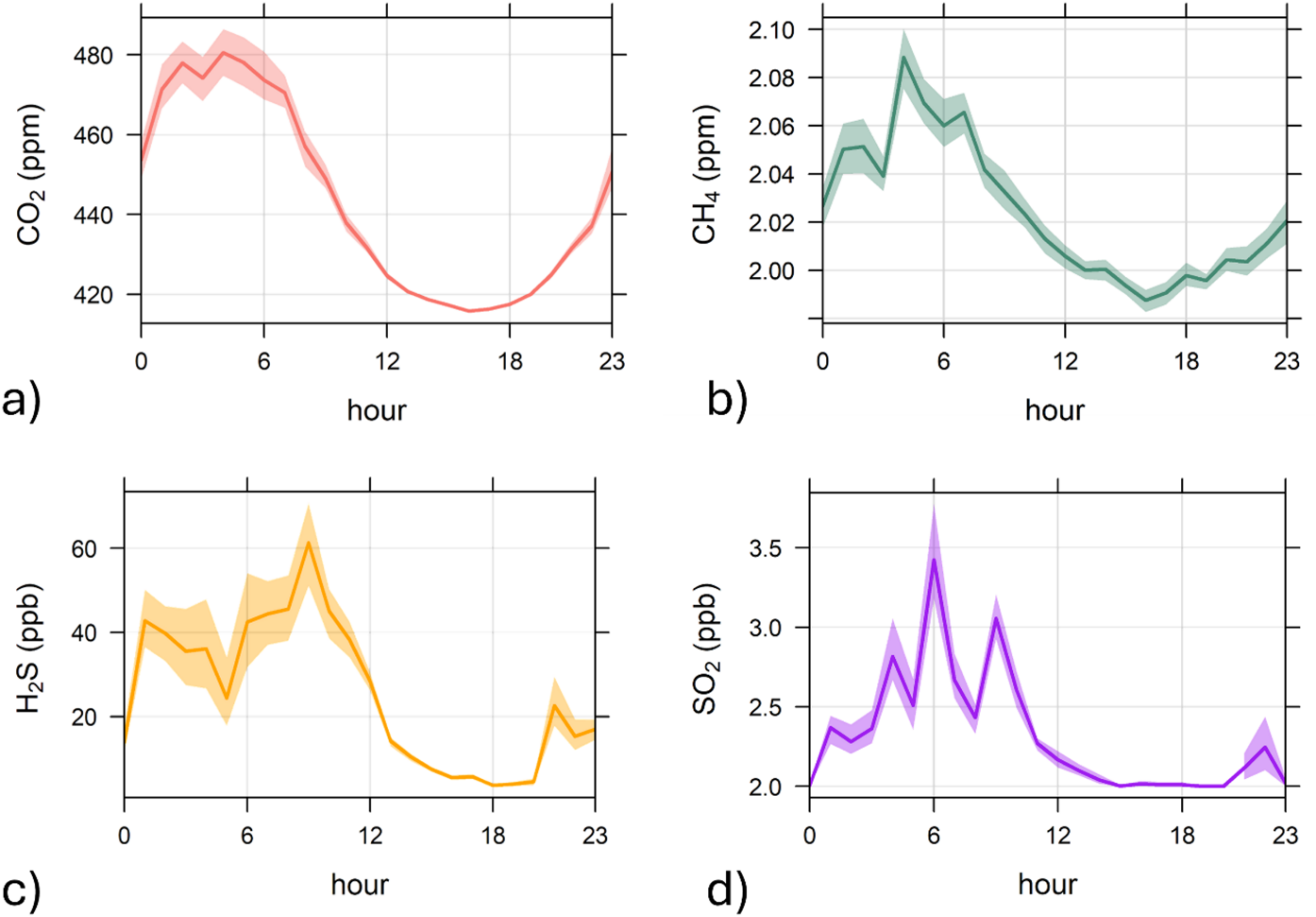
Daily variations of **a** CO_2_, **b** CH_4_, **c** H_2_S, and **d** SO_2_ concentrations

The analysis of daily variations of CO_2_, CH_4_, H_2_S, and SO_2_ concentrations (Fig. 3a–d) revealed the occurrence of relatively high concentrations during nighttime and early morning. Such temporal trends reflected the typical alternation of NBL (nocturnal boundary layer) and CBL (convective boundary layer) (Sreenivas et al., 2016; Venturi et al., 2020). Both CO_2_, rarely exceeding 550 ppm, and CH_4_ (< 2.50 ppm) concentrations (Figs. 2a and b) were lower than the alert threshold (0.5% and 0.1%, respectively; OSHA, 2019). Differently, the H_2_S concentrations occasionally exceeded the threshold value (100 ppb) recommended by WHO (2000) for exposures over 24 h (Fig. 2c).

## Discussion

### Origin of air pollutants

**Fig. 4:**
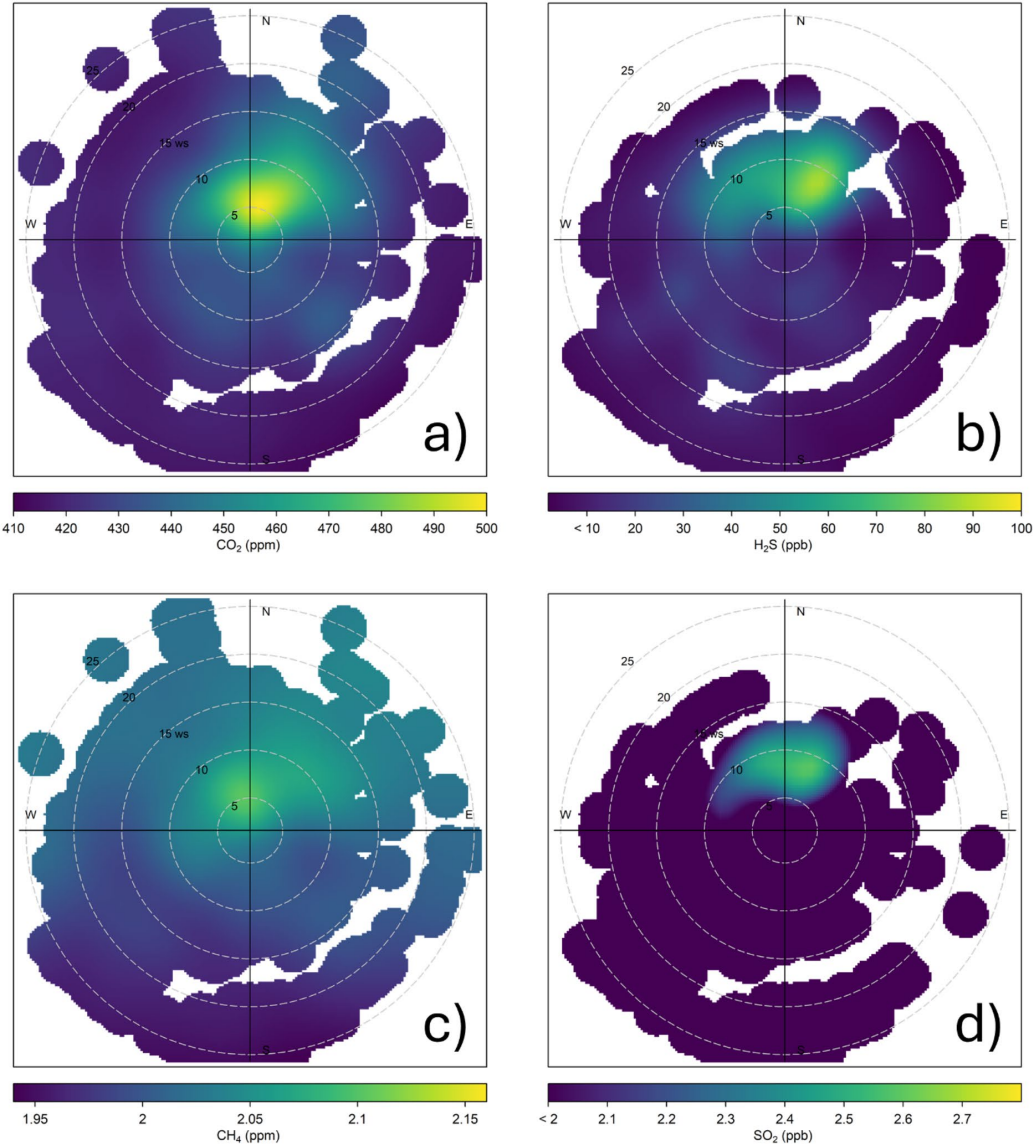
Polar plot diagrams reporting **a** CO_2_, **b** H_2_S, **c** CH_4_, and **d** SO_2_ concentrations

Relatively high CO_2_ and H_2_S concentrations were consistently associated with low-speed winds (< 10 m/s) from the northern sectors (Fig. 4a,b), where most potential contaminant sources occurred, including (i) Vehicular traffic from Tivoli Terme to the N; (ii) Hydrothermal fluid emissions from thermal springs to the N (Regina and Colonnelle lakes), and ponds and artificial lakes produced by quarries’ extraction activity to the NE-E sectors (Fig. 1b). Differently, CH_4_ concentrations in the air were always relatively low (< 2.50 ppm; Fig. 2c) even though potential sources are widespread all over the study area. The distribution of SO_2_ concentrations followed that of H_2_S, suggesting their common origin (Pearson’s r = 0.88). Specifically, H_2_S was dominantly produced at depth, being typical of gases from hydrothermal reservoirs (e.g. Giggenbach, 1996), whereas SO_2_ likely derived from H_2_S oxidation (e.g. Biagi et al., 2022) mostly occurring in air where free O_2_ is available.

**Fig. 5:**
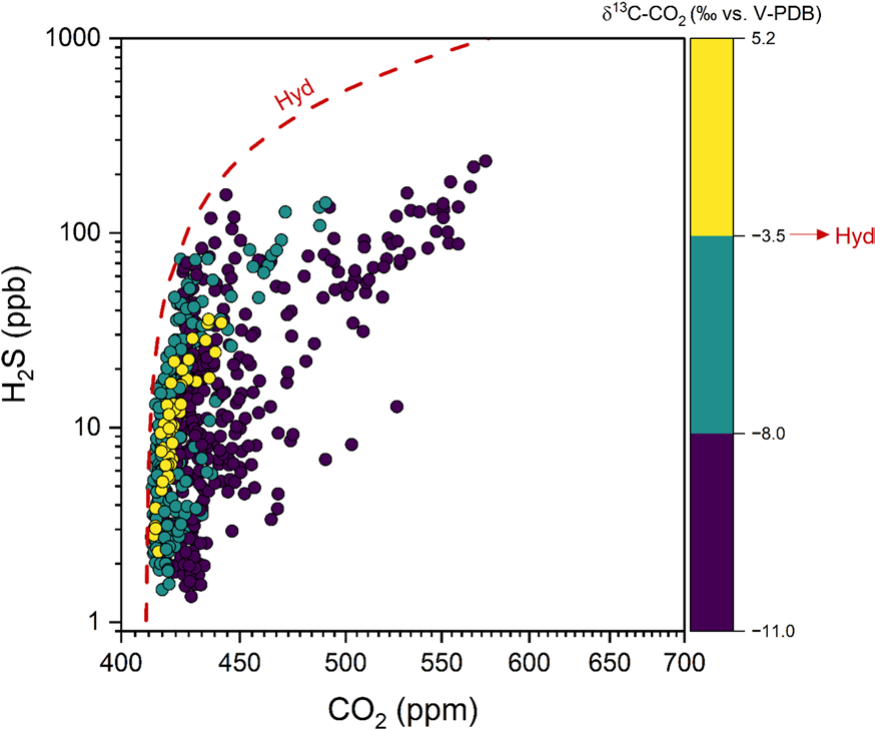
H_2_S vs. CO_2_ binary diagram. Different symbol colors are associated with δ^13^C-CO_2_ value intervals. A red line representing the dilution trend of the Tivoli hydrothermal fluid (Hyd) is also reported

The strong relationship between H_2_S and CO_2_ concentrations, the latter largely dominating the chemical composition of hydrothermal fluids (e.g. Giggenbach, 1996), was also confirmed by the H_2_S vs. CO_2_ plot (Fig. 5). Notably, most gases having δ^13^C-CO_2_ values consistent with the isotopic signature of the Tivoli thermal spring (i.e., − 3.5‰ vs. V-PDB; Minissale et al., 1997, 2002) approached the theoretical dilution trend of the hydrothermal fluids with air (Minissale et al., 1997, 2002). The slight increase in the CO_2_/H_2_S ratio relative to the hydrothermal endmember shown by these measurements was likely related to H_2_S degradation in the air (e.g., Badalamenti et al., 2001; Carapezza et al., 2003). On the other hand, the high CO_2_/H_2_S ratios measured at decreasing δ^13^C-CO_2_ values (i.e., < − 8.0‰ vs. V-PDB; Fig. 5) suggest CO_2_ addition from the typical anthropogenic activity in urban areas, e.g. gasoline combustion (δ^13^C-CO_2_ = − 27 ‰ vs. V-PDB; Venturi et al., 2020 and references therein).

**Fig. 6:**
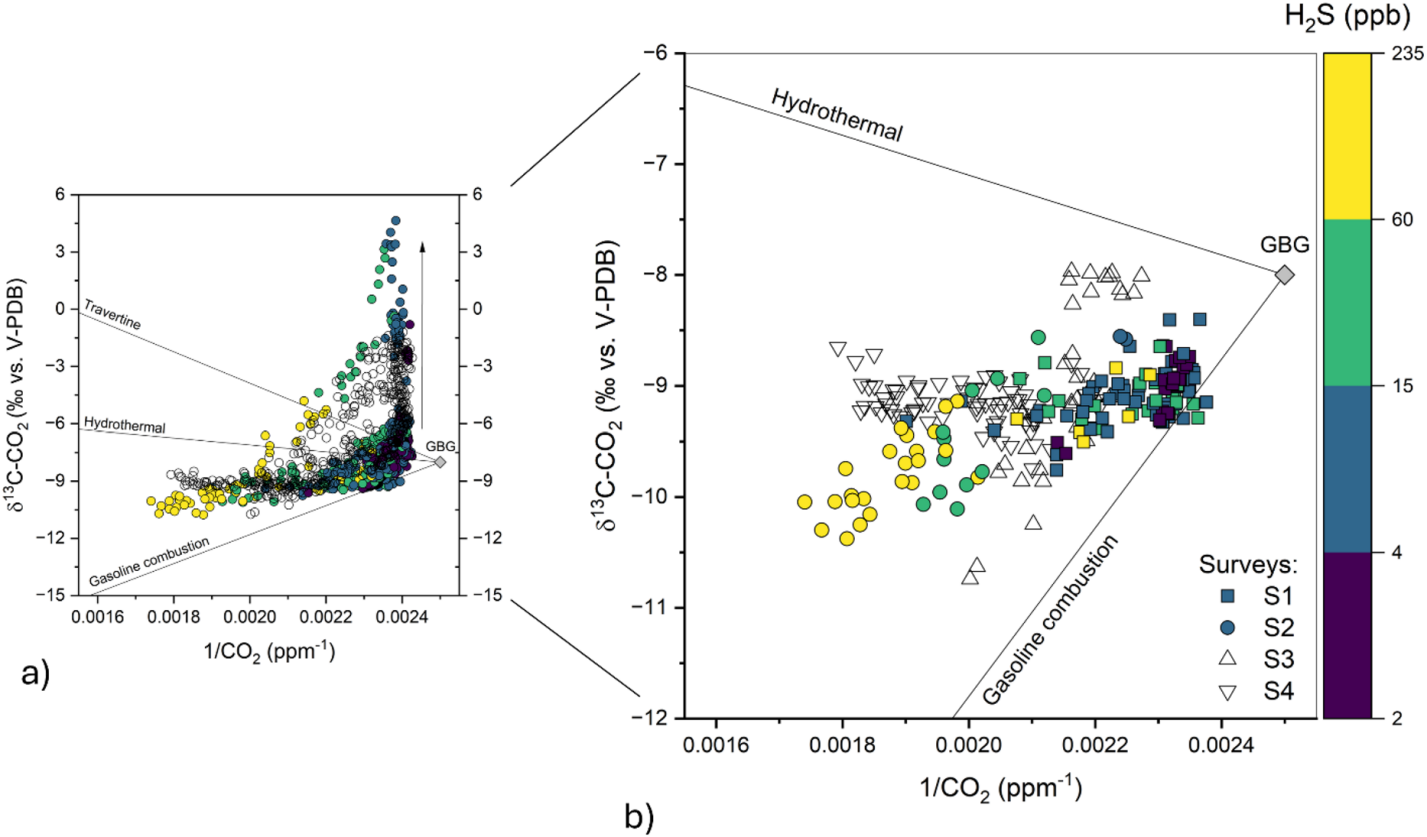
Keeling plot of CO_2_ during b the entire measuring period, and **b** nighttime divided by surveys (S1-4). Different symbol colors are associated with H_2_S concentration intervals. Theoretical mixing lines with possible sources of CO_2_ are reported. The global background values of δ^13^C-CO_2_ (GBG = − 8.5 ‰ vs. V-PDB;) and CO_2_ concentration (400 ppm) (https://www.esrl.noaa.gov/), are reported

Further insight into the main CO_2_ contaminant sources may be tentatively obtained by applying the Keeling plot analysis (Keeling, 1958, 1961). The Keeling plot analysis is based on the assumption that the measured values are the result of a two end-member mixing model between constant background air and gas from local sources during the measuring period (Pataki et al., 2003a, 2003b). Accordingly, the data analysis was conducted considering the nighttime hours (24:00–6:00) of each survey (Fig. 6b), when CO_2_ tends to accumulate in the lower atmospheric layers (Fig. 3). The estimated δ^13^C-CO_2_ values of the pure CO_2_ endmembers (− 29.6 to − 7.8‰ vs. V-PDB; Table S2) cannot be considered fully reliable, due to r^2^ values well below the commonly accepted threshold for linearity (> 0.75; Chamberlain et al., 2016) in the OLS (ordinary least squares) regression of δ^13^C-CO_2_ against 1/CO_2_. Estimates based on the highest CO_2_ concentrations, recorded on August 21st and 29th (Fig. 2a), corresponded to the most negative δ^13^C-CO_2_ values and yielded the highest r^2^ coefficients (Table S2), likely indicating a significant input from anthropogenic sources. Nevertheless, it is reasonable to assume that both anthropogenic, including gasoline combustion, and hydrothermal (− 3.5‰ vs. V-PDB; Minissale et al., 2002) sources have contributed to CO_2_ levels in the study area, influencing the dispersion of isotopic data. This hypothesis is supported by the observation that even the most negative δ^13^C-CO_2_ isotopic values (− 10.8‰ vs. V-PDB), i.e. indicating a dominant anthropogenic CO_2_ contribution, were associated with considerable H₂S concentrations (up to 234 ppb) (Fig. 6b).

**Fig. 7:**
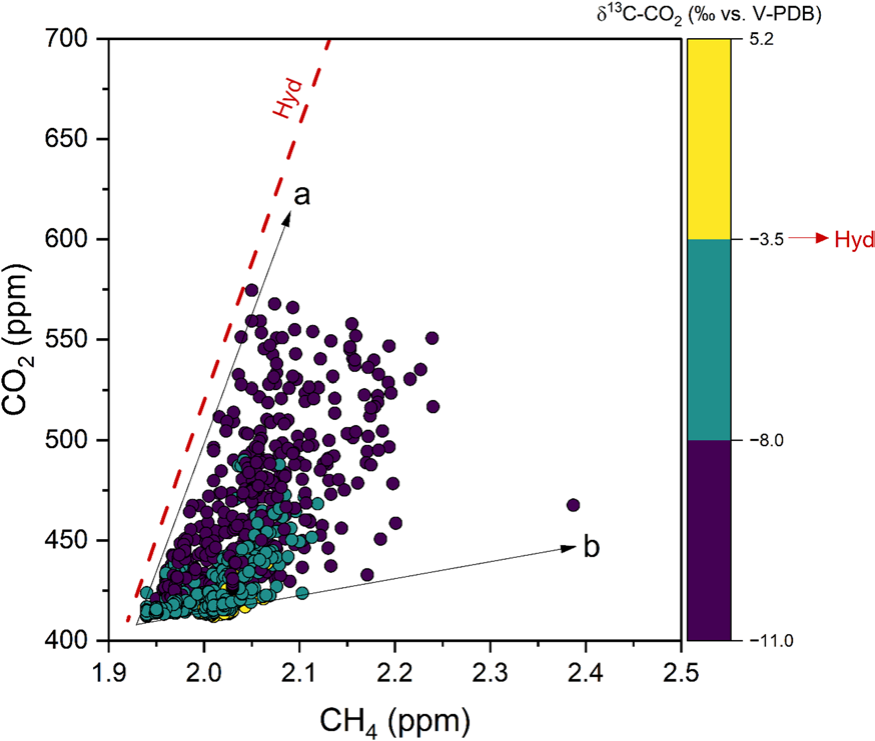
CH_4_ vs. CO_2_ binary diagram. Different symbol colors were associated with δ^13^C-CO_2_ value intervals. A red line representing the dilution trend of the Tivoli hydrothermal fluid (Hyd) is also reported

**Fig. 8:**
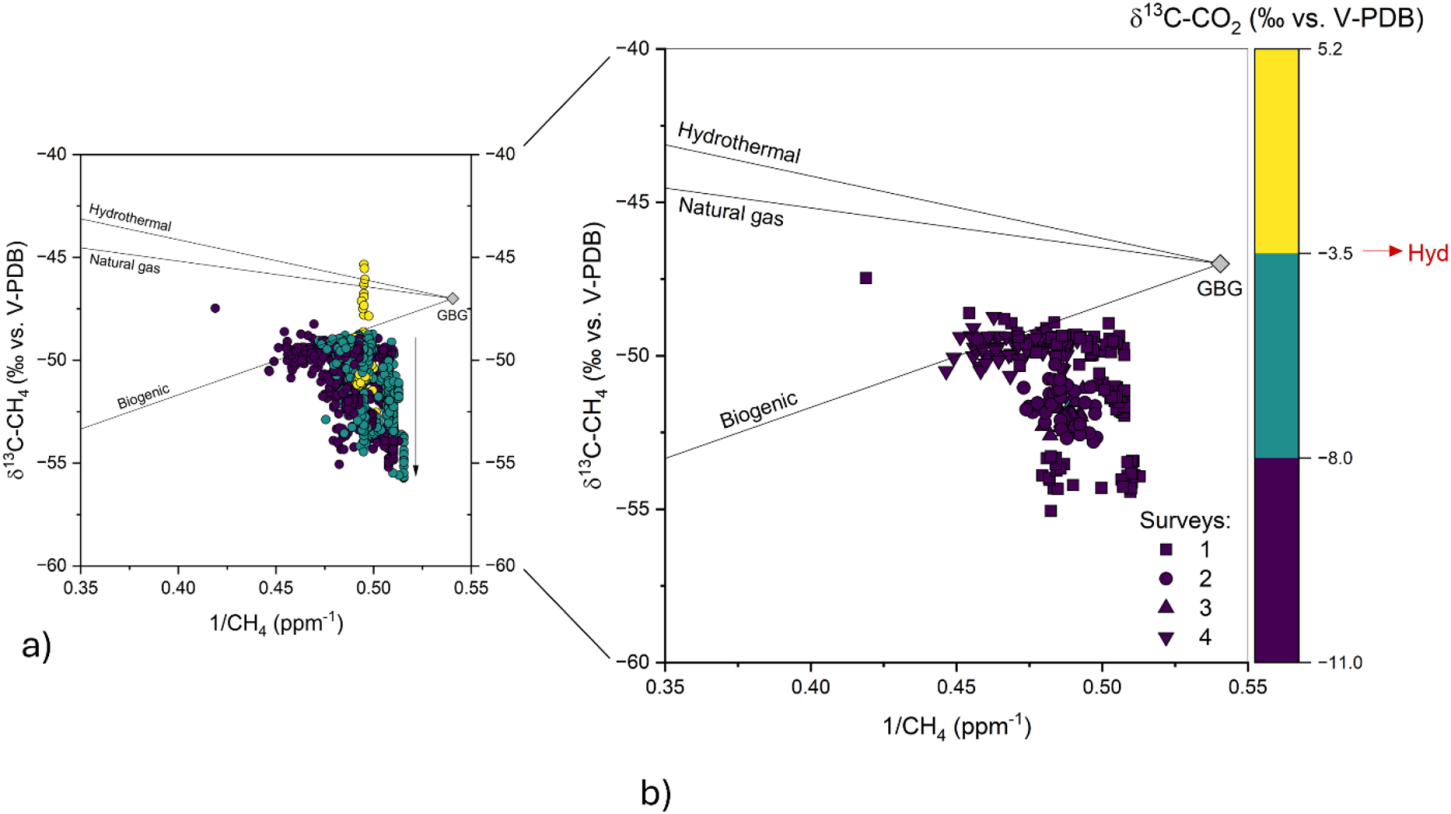
Keeling plot of CH_4_ during **a** the entire measuring period, and **b** nighttime divided by surveys (S1-4). Different symbol colors were associated with δ^13^C-CO_2_ value intervals. Theoretical mixing lines with possible sources of CH_4_ are reported. The global background values of δ^13^C-CH_4_ (-47.2‰ vs, V-PDB; Miller et al., 2002) and CH_4_ concentrations (1.85 ppm in 2017; Lan et al., 2025) are also reported

At first approximation, CO_2_ and CH_4_ were positively correlated (Fig. 7), suggesting common contaminant sources. However, their concentrations spanned between two distinct trends: trend (a) Approached the CO_2_/CH_4_ ratio of the hydrothermal gases (Minissale et al., 1997, 2002), although their δ^13^C-CO_2_ values, being mostly < − 8.0‰ vs. V-PDB, suggest that CO_2_ mostly originated from anthropogenic sources; trend (b) Had relatively low CO_2_/CH_4_ ratios, which imply contributions from CH_4_-rich sources. To shed light on the origin of CH_4_ in excess relative to the theoretical contribution of hydrothermal fluids, CH_4_ Keeling plots were constructed (Fig. 8a, b), showing that the alignment depicted by increasing CH_4_ concentrations during the nighttime of the first survey (S1, Fig. 8b) roughly pointed to a δ^13^C-CH_4_ endmember comprised between − 18.6 and − 39.4‰ vs. V-PDB (the estimate are based on data from August 6th and 7th; Table S2), in agreement with the isotopic composition of both vehicular traffic (− 32 to − 17‰ vs. V-PDB; Venturi et al., 2020) and Tivoli hydrothermal fluids (δ^13^C-CH_4_ at − 40 ‰ vs. V-PDB, unpublished data). It is worth noting that such estimations were affected by a large uncertainty, as indicated by the low values of the correlation coefficients of the regression lines (r^2^ < 0.69 and 0.45, respectively) due to the large δ^13^C-CH_4_ scattering of data having relatively low CH_4_ concentrations (Fig. 8) relative to that of the global CH_4_ background (− 47.2‰ vs, V-PDB; Miller et al., 2002). It has to be considered that the measured CH_4_ concentrations (≥ 1.94 ppm) significantly exceeded the global background value (1.85 ppm in 2021; Lan et al., 2025), a feature that is commonly observed in urban areas (e.g. Chamberlain et al., 2016; Górka et al., 2014; Nakagawa et al., 2005). Therefore, the large variation of the δ^13^C-CH_4_ values at low CH_4_ concentrations was possibly due to the occurrence of low rate CH_4_ emission from different sources (whose computed δ^13^C-CH_4_ values ranged from − 67.0 to − 18.6‰ vs. V-PDB; Table S2), such as those related to: (i) Agricultural practices (− 60 to − 55‰ vs. V-PDB; Levin et al., 1993) widespread all over the margins of the study area, and (ii) The landfill (− 50 to − 70‰ vs. V-PDB; Zazzeri et al., 2017) located to the NW relative to the measurement site (Fig. 1b). In other words, when the vertical movement of air masses was enhanced, e.g. during daytime when the soil was heated by solar irradiation, the contaminants accumulated during nighttime and early morning (Fig. 2) were not completely dispersed. Therefore, the dependence of the isotopic signature of CH_4_ on the different contaminant sources at relatively low concentrations was more evident than in periods characterized by weather conditions favoring one (or few) specific contaminant source(s). Interestingly, those data characterized by δ^13^C-CO_2_ values > − 3.5‰ vs. V-PDB showed relatively high δ^13^C-CH_4_ values (Fig. 8a), contributing to the scattering of the isotopic data at low CH_4_ concentrations. On the whole, the analysis of the CH_4_ Keeling plot (Fig. 8a, b) is scarcely helpful in assessing the origin of the occasional peaks of this gas, which may be ascribed to the different potential sources recognized in the area: the Tivoli hydrothermal fluids (δ^13^C-CH_4_ at -− 40 ‰ vs. V-PDB, unpublished data), (ii) Vehicular traffic (− 32 to − 17‰ vs. V-PDB; Venturi et al., 2020); (iii) natural gas leaks from pipelines (− 35 to − 45‰ vs. V-PDB; e.g., Venturi et al., 2020). Moreover, the δ^13^C-CH_4_ may be also controlled by isotope fractionation caused by CH_4_ oxidation in the air (e.g. Holmes, 2018).

### Background variations

It is noteworthy that as CO_2_ concentrations decreased, approaching the local background level of 412 ppm, δ^13^C values shifted well above the global δ^13^C-CO_2_ background value (− 8.5‰ vs. V-PDB; https://www.esrl.noaa.gov/) (Fig. 6a). These relatively high δ^13^C-CO_2_ values, even exceeding those of the Tivoli spring, cannot be attributed to local sources. A contribution from CO_2_-rich emissions, including hydrothermal sources from Regina and Colonnelle lakes, vehicular traffic, or the landfill, would have resulted in an increase in CO_2_ concentrations, which instead was not observed (Fig. 6a). Moreover, each of these sources should have been associated with other characteristic species, including H_2_S (and SO_2_) for hydrothermal emissions, CH_4_ for the landfill, and both CH_4_ and SO_2_ for vehicular traffic. Therefore, it is likely that the observed trend reflects an isotopic shift in the background, possibly linked to a concurrent shift in δ^13^C-CH_4_ (Fig. 8). A variable isotopic signature of background CO_2_ may possibly be ascribed to the diffuse and continuous release of ^13^C-rich CO_2_ produced by travertine deposition (Mancini et al., 2020, 2024) and, more generally, from pools and waters treated in the quarries. However, even in this case, an increase in CO_2_ concentrations would be expected.

To further investigate this phenomenon, a 24 h back-trajectory analysis of air particles was conducted using HYSPLIT models (Stein et al., 2015) at 3:00 and 15:00 each day (HTML files of the four surveys are available in the Supplementary Materials) with the splitR package in R. This aimed to assess potential changes in atmospheric circulation. While a direct causal link between air mass circulation changes and the observed isotopic shift cannot be definitively established, a notable shift in air mass origin was observed between survey 1 (Fig. S1) and survey 2 (Fig. S2), transitioning from the Atlantic-Tyrrhenian sector (NW) to the Balkan-Adriatic sector (NE). Subsequently, during surveys 3 (Fig. S3) and 4 (Fig. S4), an intermediate situation was recorded, with winds originating from both sectors. In other words, when winds originated from the Atlantic-Tyrrhenian sector (NW), background δ^13^C remained within the expected ranges (Fig. 2a, b), whereas relatively high δ^13^C values (Fig. 2a, b) were associated with air masses from the Balkan-Adriatic sector (NE).

## Conclusions

The study area, centered in the city of Tivoli where different potential natural and anthropogenic sources of air pollutants were recognized, offered a good opportunity to evaluate the information that can be gathered from chemical and isotopic parameters of C- and S-bearing gases carried out at a fixed station measurement point. This approach reproduces the strategy commonly adopted by governmental agencies for air quality control. Therefore, it represents a valuable test to evaluate the efficiency of the air monitoring stations currently operating in the country for (i) Individuating the various contaminant sources and (ii) Organizing and applying actions for their mitigation. The results of this investigation highlighted the significant air contamination caused by both natural and anthropogenic emissions. However, the measured gases (CO_2_, CH_4_, H_2_S, and SO_2_) were significantly lower than their respective threshold concentrations for outdoor air quality. The combination of isotopic and concentration data of CO_2_ and CH_4_, although promising, provided ambiguous indications regarding the effective environmental impact of the different contaminant sources. Nevertheless, the occurrence of H_2_S concentrations up to 234 ppb, i.e. well above the typical background value in uncontaminated air (< 1 ppb), undoubtedly testified that the hydrothermal fluid emissions, disseminated all around the measurement station, had a relevant environmental impact. The paper mill located to the NE of the measurement station seems to not represent a significant source of this pollutant (Fig. 6). Air quality was relatively poor when wind flew from the northern sectors relative to the measurement point and wind speed was low, favoring air stratification, whereas no significant sources of air pollutants were recognized to the S. The origin of CO_2_ and CH_4_ seems to be mostly related to anthropogenic sources, whereas the hydrothermal fluid emissions played a secondary role for these gases (Figs. 6 and 8). However, the geochemical data of this study did not allow a reliable evaluation of each specific contribution. Moreover, a shift in δ^13^C values of background CO_2_ and CH_4_ was observed, possibly linked to a change in the origin of air masses. Nevertheless, this issue remains unresolved and requires further investigation to be fully understood.

These results suggest that the typical strategy adopted for air quality assessment based on measurements carried out at one fixed monitoring station is affected by severe limitations. A unique measurement site, indeed, is too dependent on weather parameters to efficiently and continuously provide reliable information on the relative impact of the various contaminant sources occurring in a wide monitored zone. Moreover, compositional and isotopic geochemical parameters of air contaminants provide too ambiguous indications to clearly discriminate different potential sources. Considering that the high costs of the instruments able to provide such geochemical data strongly limit the number of monitoring stations that can be installed, different measurement approaches should be taken into consideration to try to solve these issues. Mobile stations may usefully integrate the concentration and isotope data measured at the fixed station (Venturi et al., 2019), allowing for periodic mapping of the distribution of air pollutants within the monitored area. Moreover, low-cost instruments (Biagi et al., 2024) that can be distributed at several sites, e.g. in the proximity of the contaminant sources potentially affecting the monitored zone and at increasing distance from them, may provide further data to control the spatial–temporal evolution of the air contaminant concentrations.

## Supplementary Information

Below is the link to the electronic supplementary material.

Supplementary file1 (R 4 kb)

Supplementary file2 (XLSX 797 kb)

Supplementary file3 (HTML 548 kb)

Supplementary file4 (HTML 519 kb)

Supplementary file5 (HTML 519 kb)

Supplementary file6 (HTML 534 kb)

Supplementary file7 (XLSX 64 kb)

Supplementary file8 (DOCX 16 kb)

## Data Availability

Data is provided within the supplementary information files.
