# Supplementary material for: Monitoring air pollutants in urbanized hydrothermal areas: challenges and benefits of traditional measurement strategies
Source: Environ Geochem Health. 2025 Mar 16;47(4):120. doi: 10.1007/s10653-025-02422-y (PMC11911267; doi:10.1007/s10653-025-02422-y)
Supplement: Supplementary file 8 — Supplementary file8 (DOCX 16 kb) [file 10653_2025_2422_MOESM8_ESM.docx]

**Table S1** δ^13^C estimations of CO_2_ and CH_4_ sources by the Keeling plot analysis.

| **Survey** | **Date** | **Intercept** | | **R²** | |
| --- | --- | --- | --- | --- | --- |
|  |  | *CO₂* | *CH₄* | *CO₂* | *CH₄* |
| S1 | Aug 05 | -7.9 | -53.1 | 0.02 | -0.03 |
|  | Aug 06 | -12.9 | -18.6 | 0.08 | 0.69 |
|  | Aug 07 | -13.5 | -39.4 | 0.32 | 0.43 |
| S2 | Aug 21 | -16.2 | -29.3 | 0.65 | 0.27 |
| S3 | Aug 29 | -29 | -67 | 0.64 | 0.02 |
| S4 | Sep 01 | -7.8 | -51.1 | 0.06 | -0.02 |
|  | Sep 02 | -8.7 | -54.8 | 0.001 | 0.1 |
